# Transcriptomic meta-analysis identifies gene expression characteristics in various samples of HIV-infected patients with nonprogressive disease

**DOI:** 10.1186/s12967-017-1294-5

**Published:** 2017-09-12

**Authors:** Le-Le Zhang, Zi-Ning Zhang, Xian Wu, Yong-Jun Jiang, Ya-Jing Fu, Hong Shang

**Affiliations:** 1 Key Laboratory of AIDS Immunology of National Health and Family Planning Commission, Department of Laboratory Medicine, The First Affiliated Hospital, China Medical University, No 155, Nanjingbei Street, Heping District, Shenyang, 110001 Liaoning Province China; 2 Collaborative Innovation Center for Diagnosis and Treatment of Infectious Diseases, Hangzhou, China

**Keywords:** HIV-1, LTNP, EC, Integrative transcriptome analyses, Microarray, Meta-analysis

## Abstract

**Background:**

A small proportion of HIV-infected patients remain clinically and/or immunologically stable for years, including elite controllers (ECs) who have undetectable viremia (<50 copies/ml) and long-term nonprogressors (LTNPs) who maintain normal CD4^+^ T cell counts for prolonged periods (>10 years). However, the mechanism of nonprogression needs to be further resolved. In this study, a transcriptome meta-analysis was performed on nonprogressor and progressor microarray data to identify differential transcriptome pathways and potential biomarkers.

**Methods:**

Using the INMEX (integrative meta-analysis of expression data) program, we performed the meta-analysis to identify consistently differentially expressed genes (DEGs) in nonprogressors and further performed functional interpretation (gene ontology analysis and pathway analysis) of the DEGs identified in the meta-analysis. Five microarray datasets (81 cases and 98 controls in total), including whole blood, CD4^+^ and CD8^+^ T cells, were collected for meta-analysis.

**Results:**

We determined that nonprogressors have reduced expression of important interferon-stimulated genes (ISGs), CD38, lymphocyte activation gene 3 (LAG-3) in whole blood, CD4^+^ and CD8^+^ T cells. Gene ontology (GO) analysis showed a significant enrichment in DEGs that function in the type I interferon signaling pathway. Upregulated pathways, including the PI3K-Akt signaling pathway in whole blood, cytokine–cytokine receptor interaction in CD4^+^ T cells and the MAPK signaling pathway in CD8^+^ T cells, were identified in nonprogressors compared with progressors. In each metabolic functional category, the number of downregulated DEGs was more than the upregulated DEGs, and almost all genes were downregulated DEGs in the oxidative phosphorylation (OXPHOS) and tricarboxylic acid (TCA) cycle in the three types of samples.

**Conclusions:**

Our transcriptomic meta-analysis provides a comprehensive evaluation of the gene expression profiles in major blood types of nonprogressors, providing new insights in the understanding of HIV pathogenesis and developing strategies to delay HIV disease progression.

**Electronic supplementary material:**

The online version of this article (doi:10.1186/s12967-017-1294-5) contains supplementary material, which is available to authorized users.

## Background

Disease progression in the absence of therapy varies significantly in HIV-infected individuals. Most patients experience progressive CD4^+^ T cell loss and develop AIDS [1]. However, a small proportion of HIV-infected patients remain clinically and/or immunologically stable for years, including long-term nonprogressors (LTNPs) who maintain normal CD4^+^ T cell counts for prolonged periods (>10 years) and elite controllers (ECs) who have undetectable viremia (<50 copies/ml) [2–4]. In past decades, numerous studies have been conducted searching for the cause for this lack of progression [5–7]. Among them, high-throughput techniques, such as microarray analysis, have contributed to the understanding of the complex host-virus interactions associated with the delayed disease progression in nonprogressors [8–12].

Using various microarray techniques and various types of samples, including whole blood, PBMC, CD4^+^ T cells, CD8^+^ T cells and monocytes, previous studies have provided valuable information on transcriptomic profiles in nonprogressors (including LTNPs and ECs, referred to as “nonprogressors” below). Several transcriptomic analyses of T cells have highlighted the role of reduced interferon-stimulated genes (ISGs) associated with the nonprogressing status in LTNPs and ECs [8, 10, 13]. T cell transcriptomic studies have also revealed the enhancing pathways in LTNPs, including the APK, WNT, and AKT pathways, contributing to cell survival and antiviral responses [14], cytokine–cytokine receptor interaction, a negative control of apoptosis or regulation of actin cytoskeleton [15]. A PBMC transcriptomic study demonstrated that cell death/proapoptotic genes were mostly downregulated and cell survival/antiapoptotic genes or genes belonging to the canonical Wnt/beta-catenin signaling pathway were upregulated in LTNPs [16]. A monocyte transcriptomic study revealed upregulation of Toll-like receptor (TLR) signaling with subsequent downregulation of MAPK, NF-kB, JAK-STAT, and the IRF cascades in LTNPs [17]. A transcriptomic study on whole blood from LTNPs and progressors identified a novel ISG gene, LY6E, which restrains the hyperactivation of monocytes during HIV-1 infection [11].

Although the accumulating transcriptome data provide useful information in studying the host protective immune responses in nonprogressors, the identification of key genes and pathways from these studies was restricted due to the limited sample size in the independent study. A transcriptome meta-analysis can incorporate high-throughput data from multiple independent studies and overcome the aforementioned limitations. In addition, different cells have unique functional activities in the immune responses of nonprogressors, but the differences in the transcriptomic profiles between the major cell types of nonprogressors have not been elucidated. In the present study, we performed a meta-analysis of five independent microarray datasets to identify differentially expressed genes (DEGs) in whole blood, CD4^+^ and CD8^+^ T cells from HIV-infected nonprogressors compared with progressors. Our study provides a comprehensive evaluation of the gene expression profiles in the major blood types of nonprogressors, which will provide new insights in the understanding of HIV pathogenesis and developing strategies to delay HIV-1 disease progression.

## Methods

### Microarray data collection

The gene expression microarray datasets with the keywords “long-term nonprogressor” and “elite controller” were downloaded from Gene Expression Omnibus (NCBI) database [18]. LTNPs or ECs were considered “case group” while progressors, including rapid progressors (RPs), chronic progressors (CPs) or normal progressors (NPs) were considered “control group”. Data sets with a sample source other than whole blood, CD4^+^ and CD8^+^ T cells from nonprogressors were excluded. Five independent microarray datasets with raw data were selected and the details about these datasets are outlined in Table 1. The following information was extracted from each of the studies that were selected: GEO accession; numbers of patients and controls; sample source; platform and gene expression data. Of the five datasets, three were conducted in Affymetrix HG U133 GeneChips while the others were performed in the Illumina beadchip platform. We compared microarray data for LTNP/ECs (n = 81) and progressors (n = 98) from the public database submissions. Two datasets included the transcriptome profiles of whole blood from 20 nonprogressors and 18 progressors. Two datasets included CD4^+^ T cells from 19 nonprogressors and 32 progressors. Three datasets included CD8^+^ T cells from 42 nonprogressors and 48 progressors.

**Table 1:** Summary of transcriptome datasets used in this study

Study	GEO accession	Sample size		Sample source	Platform
		LTNP case	Control		
1	GSE56837	15	11	Whole blood	GPL6884, Illumina HumanWG-6 v3.0 expression beadchip
2	GSE57730	5	7	Whole blood	GPL570, Affymetrix Human Genome U133 Plus 2.0 Array
3	GSE28128	14	27	CD4^+^ T cell	GPL6884, Illumina HumanWG-6v3.0 expression beadchip
4	GSE28128	13	25	CD8^+^ T cell	GPL6884, Illumina HumanWG-6v3.0 expression beadchip
5	GSE6740	5	5	CD4^+^ T cell	GPL96, Affymetrix Human Genome U133A Array
6	GSE6740	5	5	CD8^+^ T cell	GPL96, Affymetrix Human Genome U133A Array
7	GSE24081	24	18	CD8^+^ T cell	GPL3921, Affymetrix HT Human Genome U133 Array

### Analysis of differential gene expression

To find DEGs in different types of samples between nonprogressors and progressors, the data collected from each eligible microarray study were imported to the INMEX (integrative meta-analysis of expression data) program (http://www.inmex.ca/INMEX/) [19] to carry out the meta-analysis. The data were annotated after converting the gene and probe IDs to the corresponding Entrez IDs. The intensity values for each probe set were log_2_ transformed then uploaded, processed, and annotated for data integrity. Batch effect correction option (ComBat) was used to reduce potential batch effect [20]. After a data integrity check, we carried out a meta-analysis using the combined rank orders method whose implementation is based on the RankProd package [21]. The whole process was repeated 100 times to compute the p value and false discovery rate (FDR) associated with each gene [19]. The selected genes are sorted based on the combined rank product (combined RP). The lower the combined RP value, the more significant the candidate gene is for heterologous expression [22]. Statistical analyses were performed using the INMEX program [19]. A p value <0.05 was considered statistically significant in the analysis.

### Gene ontology terms and pathway enrichment

Functional interpretation (gene ontology analysis and pathway enrichment analysis) of the DEGs identified in this meta-analysis was performed with Database for Annotation, Visualization and Integrated Discovery (DAVID, Version 6.8 Beta), a web-based tool for Gene Ontology (GO) enrichment analysis (http://david.abcc.ncifcrf.gov/) [23]. Gene symbol lists were uploaded and analyzed using functional annotation chart for GO Biological Process Annotation (GO_BP) and Kyoto Encyclopedia of Genes and Genomes (KEGG) Pathway. Representative GO Biological Process terms and KEGG pathways selected from the top significantly enriched charts are reported in the figures. A Benjamini-corrected p value less than 0.05 was used to identify a statistically significant analysis.

## Results

### DEGs in whole blood, CD4^+^ and CD8^+^ T cells from nonprogressors

First, we identified the DEGs in whole blood from nonprogressors. According to the results of our meta-analysis, 1160 genes in whole blood were identified to be differentially expressed between nonprogressors and progressors. Among the 1160 DEGs, 215 genes were upregulated and 945 genes were downregulated. The downregulated genes included genes that have been reported to be associated with HIV-1 disease progression or pathogenesis, such as CD38 and some ISGs [11, 13, 24].

There were 854 DEGs in CD4^+^ T cells between nonprogressors and progressors across microarray datasets. Among the 854 DEGs, 310 genes were upregulated and 544 genes were downregulated. The downregulated genes included ISGs, such as IFI44L, MX1 and IFI27.

There were 1319 genes in CD8^+^ T cells identified to be differentially expressed between nonprogressors and progressors across microarray datasets. Among the 1319 DEGs, 524 genes were upregulated and 795 genes were downregulated. The downregulated genes also included CD38 and some ISGs. The top 20 most significantly upregulated and downregulated DEGs in different types of cells in the comparison of nonprogressors and progressors are shown in Additional file 1: Table S1A–C. The DEGs in different types of cells are shown in Additional file 2: Table S2A–C.

**Fig. 1:**
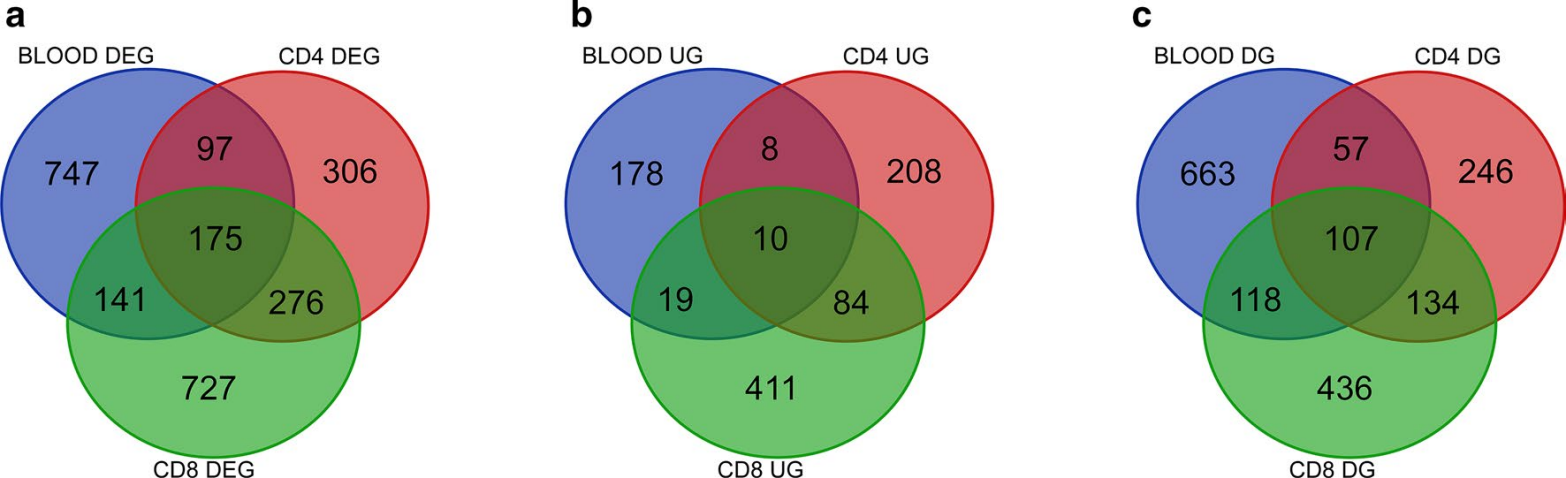
Venn diagram representing the number of DEGs in nonprogressors compared with progressors in whole blood (blue circle), CD4^+^ T cells (red circle) and CD8^+^ T cells (green circle). The overlapping area indicates DEGs identified in the three types of samples. *UG* upregulated genes. *DG* downregulated genes. **a** All the DEGs found in the three types of samples. **b** The upregulated genes found in the three types of samples. **c** The downregulated genes found in the three types of samples

Second, we investigated whether some of the genes were shared in the comparison of different types of samples between nonprogressors and progressors. The Venn diagram showed that 175 DEGs were significantly altered in all three sample types. Among them, ten upregulated DEGs and 107 downregulated DEGs showed the same variation trend in all the three sample types (Fig. 1; Additional file 3: Table S3A and B). Ten upregulated DEGs and the top 20 most significantly downregulated DEGs shared in the three sample types are shown in Table 2. The upregulated DEGs included CCR7, and the downregulated DEGs involved some important ISGs, CD38 and co-inhibitory molecule LAG-3.

**Table 2:** The 10 most significantly up-regulated and 20 most significantly down-regulated genes in nonprogressors of the three types of samples

Entrez ID	Gene symbol	Combined rank product	Average log fold change
	10 most significantly up-regulated genes		
1236	CCR7	693.97	0.087221
4753	NELL2	1075.15	0.267438
23531	MMD	1092.11	0.317429
6653	SORL1	1446.07	0.192771
54855	FAM46C	1718.18	0.210897
55544	RBM38	1942.46	0.220304
7057	THBS1	2070.14	0.187822
10320	IKZF1	2090.63	0.055676
9882	TBC1D4	2157.42	0.127719
55893	ZNF395	2458.17	0.091132
	20 most significantly down-regulated genes		
3429	IFI27	35.61	−0.948905
9636	ISG15	259.82	−0.534496
4061	LY6E	260.35	−0.607156
26010	SPATS2L	278.23	−0.519912
26519	TIMM10	289.63	−0.804127
10964	IFI44L	296.89	−0.427484
9381	OTOF	360.09	−0.464578
6036	RNASE2	421.70	−0.442343
952	CD38	562.80	−0.723494
3902	LAG3	618.23	−0.503929
644	BLVRA	623.86	−0.523236
81030	ZBP1	700.78	−0.423206
3512	IGJ	704.39	−0.522450
3665	IRF7	762.54	−0.566041
9997	SCO2	982.28	−0.652977
7298	TYMS	1192.70	−0.491925
3965	LGALS9	1449.29	−0.420535
10549	PRDX4	1557.82	−0.435958
10287	RGS19	1709.44	−0.423876
3113	HLA-DPA1	1992.11	−0.496091

### DEG functional classification and pathway assignment

**Fig. 2:**
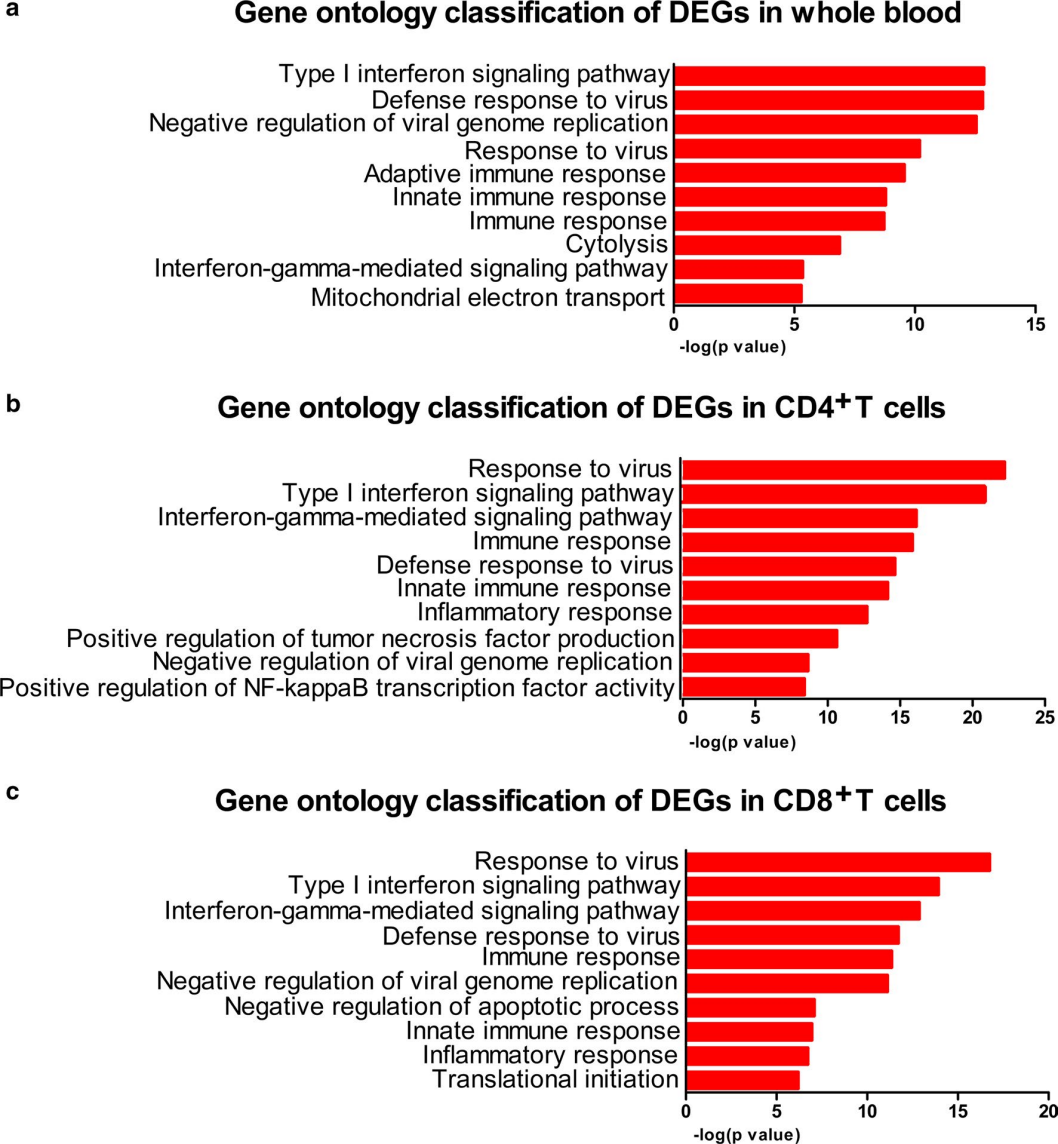
The gene ontology (GO) analysis of DEGs in nonprogressors. **a** The 10 most significantly enriched GO terms for whole blood. **b** The 10 most significantly enriched GO terms for CD4^+^ T cells. **c** The 10 most significantly enriched GO terms for CD8^+^ T cells. p < 0.05 and FDR < 0.01 were used as the threshold for GO analysis

Gene ontology analysis and KEGG pathway analysis were carried out for the functional investigation of DEGs. In the GO analysis, “type I interferon signaling pathway”, “interferon-gamma-mediated signaling pathway” and “defense response to virus” were significantly enriched for the DEGs in all three sample types (Fig. 2a–c). Additionally, the 10 most significantly enriched GO terms for DEGs in CD8^+^ T cells included “negative regulation of apoptotic process”, “inflammatory response” and “translational initiation” (Fig. 2c).

**Fig. 3:**
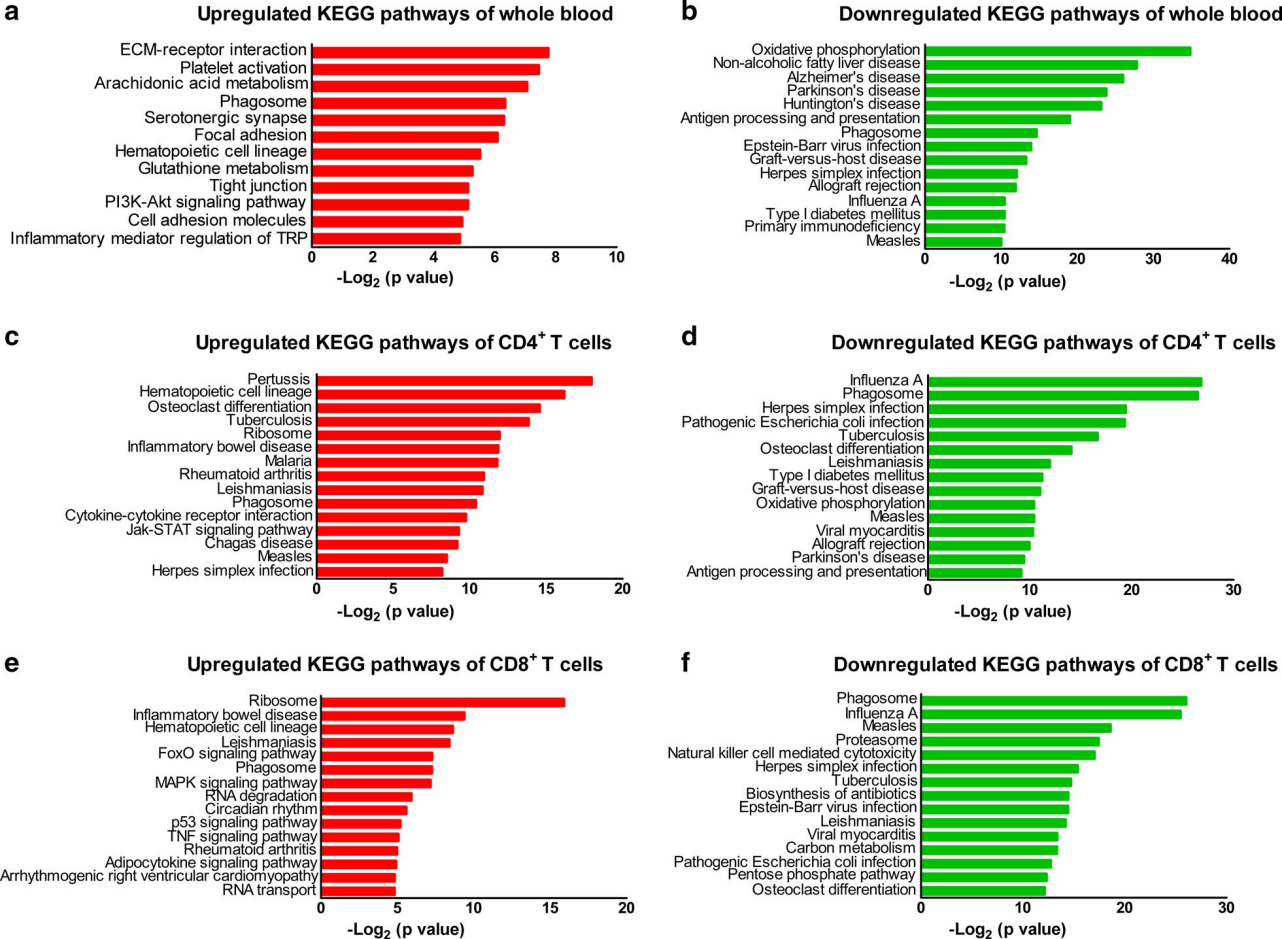
KEGG pathways of the upregulated and downregulated DEGs in nonprogressors. **a**, **b** The 15 most significantly upregulated pathways and 15 most significant downregulated pathways of whole blood. **c**, **d** The 15 most significantly upregulated and 15 most downregulated pathways of CD4^+^ T cells. **e**, **f** The 15 most significantly upregulated and 15 most downregulated pathways of CD8^+^ T cells. p < 0.05 and FDR < 0.01 were used as the threshold for KEGG pathway assignment

In the pathway analysis, we identified the top 15 most significantly upregulated and downregulated pathways for DEGs in different sample types in the comparison between nonprogressors and progressors (Fig. 3a–f). Oxidative phosphorylation (OXPHOS) was significantly downregulated in whole blood (1 top, Fig. 3b), CD4^+^ T cells (10 top, Fig. 3d) and CD8^+^ T cells (53 top, data not shown). Antigen processing and presentation were significantly downregulated in both whole blood and CD4^+^ T cells. In addition, pathways including PI3K-Akt signaling pathway in whole blood, cytokine–cytokine receptor interaction and Jak-STAT signaling pathway in CD4^+^ T cells and MAPK signaling pathway in CD8^+^ T cells were identified.

### Metabolic pathways associated with HIV nonprogressors

**Fig. 4:**
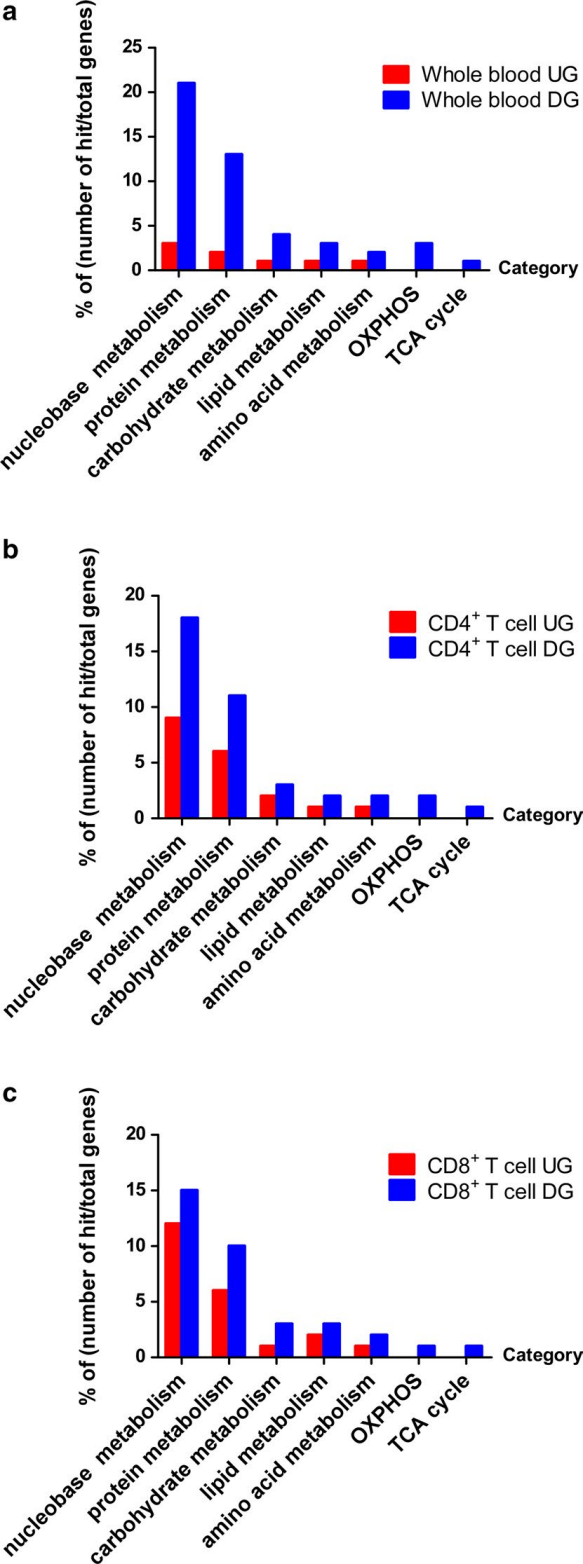
GO classification of DEGs specific to whole blood (**a**), CD4^+^ (**b**) and CD8^+^ T cells (**c**). A total of 1160 whole blood-specific, 854 CD4^+^ T cell-specific and 1319 CD8^+^ T cell-specific DEGs were categorized based on GO slim classification (p < 0.05). A bar represents each gene ontology category. The height of the bar represents the percentage of genes observed in each category. The percentage of genes per category is indicated upon the bars text. *UG* upregulated genes. *DG* downregulated genes

It has become increasingly clear that immune function is intimately linked to metabolic programs [25, 26]. Metabolic pathways have been reported to play important roles in the pathogenesis of HIV [27, 28]. After we identified that OXPHOS was downregulated in all kinds of samples in nonprogressors compared with progressors, we then performed the GO metabolism analysis with the upregulated and downregulated DEGs to investigate the alterations of metabolic pathways in nonprogressors. We found that there are more downregulated DEGs than upregulated DEGs in “carbohydrate metabolic process”, “cellular amino acid metabolic process”, “lipid metabolic process”, “nucleobase-containing compound process”, “protein metabolic process”, “TCA cycle” and “OXPHOS” in whole blood, CD4^+^ and CD8^+^ T cells (Fig. 4a–c). Nearly all DEGs in OXPHOS and the TCA cycle were downregulated in the three kinds of samples in the comparison of nonprogressors and progressors.

## Discussion

Identification of the most relevant genes and pathways involved in HIV-infected nonprogressors is important in understanding the molecular and cellular processes determining the cause for the nonprogression status in these patients. In this study, we performed a meta-analysis of multiple public microarray datasets to investigate the transcriptomic profiles of HIV-infected nonprogressors, including LTNPs and ECs. Through transcriptomic meta-analysis, we identified DEGs in various types of samples in the comparison of nonprogressors and progressors, including whole blood, CD4^+^ and CD8^+^ T cells.

We found that the expression of multiple important ISG genes, CD38 and LAG-3 was significantly downregulated in HIV-infected nonprogressors compared with that of progressors in all the three sample types. The downregulation of important ISG genes, including LY6E, IFI27, ISG15 and IFI44L, was observed in our study. Type-1 interferons (IFNs) is of critical importance for its potent antiviral effects [29]. However, it is well established that type-1 IFNs exert their full antiviral effect at very low concentration and their expression is required locally [30, 31]. Emerging lines of evidence reveal that high level and sustained type-1 IFNs expression is associated with hyper-immune activation and disease progression in persistent infections [31–34]. Dynamics of type-1 IFNs distinguishes Simian immunodeficiency virus (SIV) infection of natural hosts, that do not develop AIDS, from pathogenic SIV infections [35–38]. Natural hosts rapidly mute their type-1 IFNs responses after acute SIV infection whereas disease-susceptible macaque species maintain type-1 IFNs signaling indefinitely, which triggers hyper-activation of immune system and contributes to provide an environment that favors progression to AIDS [29, 36–39]. As the primary producers of type-1 IFNs, plasmacytoid dendritic cells (pDCs) in natural hosts of SIV have attenuated recruitment to lymphoid tissues compared with non-natural host [39–41]. This reduces pDCs exposure to sites of high level virus replication, and limits the pathogenic T-cell activation and chronic inflammation driven by activated pDCs [39, 41, 42]. In HIV infected human, although pDCs are depleted in blood during chronic infection, they accumulate in lymph nodes [39, 41–43], which may directly contribute to triggering sustain pathogenic immune activation [41–45]. High levels of IFN-α in the serum of chronically HIV-infected patients, as well as in tonsils during both chronic and acute HIV infection were observed [46, 47], which positively correlated with markers of immune activation [48, 49]. The expression of ISGs is elevated in HIV-infected rapid progressors rather than in nonprogressors [9–11, 13, 14]. Our results indicated that reduced expression of ISGs, which leads to reduced activation of immune system, was a key factor affecting disease progression. This was confirmed by our further finding that CD38 was downregulated in HIV-infected nonprogressors. CD38 is an activation marker and a nicotinamide adenine dinucleotide consuming enzyme (NADases) [15, 50]. Previous studies showed that the elevated expression of CD38 has a strong relationship with activation and cell aging [15, 50]. Our results showed that nonprogressors have lower expression of CD38 in different types of cells, which was beneficial in maintaining the survival of the cell. In addition, we found a lower expression of LAG-3, an important coinhibitory molecule in the immune system, in HIV-infected nonprogressors. It has been reported that LAG-3 is associated with immune dysfunction/exhaustion of T cells [51]. Taken together, our data revealed that HIV-infected nonprogressors have lower expression of ISGs, CD38 and LAG-3, which was beneficial in maintaining a healthy status of the immune system and contributed to the nonprogression of the disease.

Gene ontology analysis and pathway enrichment analysis offered insight into the possible roles of DEGs in the pathogenesis of nonprogressors. Consistent with our data on DEGs, we found that the type I interferon signaling pathway was involved in the most significantly enriched terms shared by the three sample types. Furthermore, we found an upregulated PI3K-Akt signaling pathway in whole blood and MAPK pathway in CD8^+^ T cells in nonprogressors. Both pathways contribute to cell survival and a successful immune response depends upon the ability of T lymphocytes to respond to antigenic stimulation [14, 15, 52]. In addition, the MAPK pathway plays an important role in T cell homeostasis [53], which is required for the cytotoxic activity of most CD8^+^ T cells [54]. Wu et al. reported that the nonprogressing status in HIV-infected LTNPs was associated with the MAPK, WNT, and AKT pathways. Our results are consistent with their study, indicating that the upregulated signaling pathways in nonprogressors is helpful for cell survival and the development of effector functions of the immune system.

In the past several years, a wealth of evidence has emerged illustrating how metabolism supports many aspects of immune system biology [55]. The field of cellular immunometabolism has made big strides over the past decade, becoming one of the hottest areas of research in immunology [56]. Through KEGG Pathway analysis, we found OXPHOS was downregulated in all kinds of samples, including whole blood, CD4^+^ and CD8^+^ T cells. Through further analysis of metabolic pathways, we found the number of downregulated DEGs was more than upregulated DEGs in different metabolic processes, including carbohydrate metabolic process, cellular amino acid metabolic process, lipid metabolic process and protein metabolic process, and almost all the DEGs in OXPHOS and the TCA cycle were downregulated in the three sample types in nonprogressors compared with progressors. Previous studies have indicated that HIV itself and/or ART can damage the mitochondria, affecting the normal functioning of the cell [57–59]. Two recent studies showed that HIV infection caused the upregulation of components of OXPHOS, TCA cycle, amino acid metabolism, and fatty acid metabolism in human CD4^+^ T cell lines at the protein level, which may be compensation for the mitochondria dysfunction [60, 61]. Previous studies have demonstrated that the LTNPs had slighter mitochondrial impairment and lower frequencies of cells with decreased mitochondrial membrane potential; this correlates may result in suppression of spontaneous apoptosis and higher CD4^+^ T cells counts when compared to HIV infected individuals [44, 45]. Compared with viremic patients in the HAART (VIR) group, LTNP downregulated the OXPHOS pathway and the TCA cycle in CD4^+^ and CD8^+^ T cells [14, 62]. Our results revealed that nonprogressors have lower levels of the OXPHOS pathway and the TCA cycle, which may be due to the milder mitochondrial impairment in nonprogressors than progressors. Generating more effective adoptive cellular immunotherapies to rescue the abnormal metabolic profiles in progressors is valuable for delaying disease progression.

## Conclusions

In summary, our meta-analysis of microarray studies provided an overview of the differential gene expression signatures in HIV-infected nonprogressors compared to progressors. We revealed that nonprogressors had reduced expression of type I interferon signaling pathway and associated genes. Downregulated DEGs in OXPHOS and the TCA cycle were also observed, which may be a compensation of mitochondrial dysfunction in HIV infection. Our meta-analysis will facilitate the understanding of unique transcriptomic profiles in HIV-infected nonprogressors and provide information for immune intervention of the disease progression of HIV.

## Additional files

**Additional file 1: Table S1A.** The 20 most significantly up-regulated and 20 most significantly down-regulated genes in nonprogressors. **Table S2B.** The 20 most significantly up-regulated and 20 most significantly down-regulated genes in nonprogressors of CD4^+^ T cells. **Table S3C.** The 20 most significantly up-regulated and 20 most significantly down-regulated genes in nonprogressors of CD8^+^ T cells.

**Additional file 2: Table S2A.** All the DEGs in nonprogressors of whole blood. **Table S2B.** All the DEGs in nonprogressors of CD4^+^ T cells. **Table S3C.** All the DEGs in nonprogressors of CD8^+^ T cells.

**Additional file 3: Table S3A.** All the DEGs in nonprogressors of the three types of samples. **Table S3B.** All the down-regulated DEGs in nonprogressors of the three types of samples.

## Abbreviations

HIV-1: human immunodeficiency virus 1
AIDS: acquired immunodeficiency syndrome
LTNP: long-term nonprogressor
EC: elite controller
NP: normal progressor
CP: chronic progressor
RP: rapid progressors
ART: antiretroviral therapy
DEGs: differentially expressed genes
DAVID: database for annotation, visualization and integrated discovery
GEO: gene expression omnibus
GO: gene ontology
KEGG: kyoto encyclopedia of genes and genomes
LAG-3: lymphocyte activation gene 3
ISG: interferon-stimulated gene
ISG15: interferon-stimulated gene 15
IFI44: interferon induced protein 44
IFI44L: interferon induced protein 44 like
OXPHOS: oxidative phosphorylation
TCA: tricarboxylic acid
IFNs: interferons
SIV: Simian immunodeficiency virus
pDCs: plasmacytoid dendritic cells
